# Breakthrough COVID-19 in Vaccinated Patients with Haematologic Malignancies—The First Single-Centre Experience from the Czech Republic

**DOI:** 10.3390/life12081184

**Published:** 2022-08-03

**Authors:** Martin Čerňan, Tomáš Szotkowski, Jiří Minařík, Milan Kolář, Pavel Sauer, Vojtěch Látal, Jana Zapletalová, Tomáš Papajík

**Affiliations:** 1 Department of Haemato-Oncology, Faculty of Medicine and Dentistry, Palacky University and University Hospital Olomouc, I.P. Pavlova 6, 77 900 Olomouc, Czech Republic; tomas.szotkowski@fnol.cz (T.S.); abretina@seznam.cz (J.M.); latalvojtech@seznam.cz (V.L.); tomas.papajik@fnol.cz (T.P.); 2 Department of Microbiology, Faculty of Medicine and Dentistry, Palacky University and University Hospital Olomouc, Hněvotínská 3, 77 515 Olomouc, Czech Republic; milan.kolar@fnol.cz (M.K.); pavel.sauer@fnol.cz (P.S.); 3 Department of Medical Biophysics, Faculty of Medicine and Dentistry, Palacky University Olomouc, Hněvotínská 3, 77 515 Olomouc, Czech Republic; ja.zapletalova@upol.cz

**Keywords:** COVID-19, SARS-CoV-2, vaccination, haematological malignancy, mortality, monoclonal antibodies

## Abstract

Vaccination is an important tool in the fight against the COVID-19 pandemic in patients with haematologic malignancies. The paper provides an analysis of the course of breakthrough SARS-CoV-2 infection in a group of vaccinated patients with haematological malignancy and a comparison with a historical cohort of 96 non-vaccinated patients with haematologic malignancies and bone marrow failure syndromes (two patients) in the treatment of COVID-19. A severe or critical course of COVID-19 was significantly less frequent in the group of vaccinated patients (10.2% vs. 31.4%, *p* = 0.003). The need for hospitalisation due to COVID-19 was significantly lower in vaccinated patients (27.1% vs. 72.6%, *p* < 0.0001) and the duration of hospitalisation was significantly shorter (10 vs. 14 days, *p* = 0.045). Vaccinated patients were insignificantly less likely to require oxygen therapy during infection. COVID-19 mortality was significantly higher in non-vaccinated patients (15.6% vs. 5.1%, *p* = 0.047). The paper demonstrated a significant positive effect of vaccination against COVID-19 on a less severe clinical course of infection, lower need for hospitalisation and mortality. However, the results need to be evaluated even in the context of new antivirals and monoclonal antibodies against SARS-CoV-2 or virus mutations with different biological behaviour.

## 1. Introduction

SARS-CoV-2-induced COVID-19 represents a major infectious complication in patients with haematologic malignancies. The first cases of the disease were reported in 2019 in Wuhan, China, followed by the spread of the virus worldwide with a global pandemic that has lasted to the present day [1]. Published studies have demonstrated a significantly higher mortality rate in patients with haematologic malignancies compared with patients with solid tumours and the general population [2,3,4]. Several risk factors of a complicated infection course have been described—older age of the patients, comorbidities, ongoing anticancer therapy, and uncontrolled underlying tumour disease [2,4,5]. The study by Pagano et al. comprising 3801 cases of COVID-19 among patients with haematologic malignancies from 132 centres in 32, mostly European, countries showed infection to be severe/critical in 63.8% cases, pointing out the severity of the pandemic. The lymphoproliferative diseases were mainly represented by non-Hodgkin lymphoma, multiple myeloma, and chronic lymphocytic leukaemia, whereas myeloproliferative malignancies were mainly represented by acute myeloid leukaemia and myelodysplastic syndromes [5]. The highest infection mortality rate was observed in studies in patients with acute leukaemias and bone marrow failure syndromes [5,6]. Ongoing research of the biology of the virus combined with the development of new treatment modalities has enabled the gradual introduction of monoclonal antibodies and new antivirals into clinical practice after corticosteroids, convalescent plasma, and remdesivir, leading to a reduction in mortality in infected patients [7]. A major breakthrough in the fight against the pandemic was the introduction of vaccination against COVID-19. Published papers have suggested a poorer antibody response in patients with haematologic malignancies in comparison with patients with solid tumours or the general population [8]. Lymphodepletion treatment, especially anti-CD20 antibodies, had an adverse effect on the antibody response (seroconversion) [8,9,10]. Despite generally lower immunogenicity of vaccines in the population of patients with haematologic malignancies, vaccination has led to a reduction in COVID-19 mortality as compared to non-vaccinated patients [11]. A study from the United States by the National COVID Cohort Collaborative Consortium among 6860 cases of breakthrough COVID-19 identified vaccinated patients with solid tumours and haematologic malignancies to be at a significantly higher risk of infection and severe outcomes compared with noncancer patients. Compared with solid tumours, haematologic malignancies were at increased risk for breakthrough infections [12].

The aim of the presented paper was to analyse the course of SARS-CoV-2 infection in the population of patients with haematologic malignancies who underwent vaccination and to compare the results with our own historical cohort of patients from the period before the introduction of vaccination into routine practice. The purpose was to bring the first single-centre experience from the Czech Republic with haematological malignancy patients who experienced breakthrough infection following COVID-19 vaccination.

## 2. Patients and Methods

### 2.1. Study Description

The paper is a retrospective descriptive analysis of a group of patients with haematological malignancy treated at the Department of Haemato-Oncology, Faculty of Medicine and Dentistry, Palacky University and University Hospital Olomouc in the period from 1 January 2021 to 31 January 2022, with confirmed SARS-CoV-2 infection, despite full vaccination against COVID-19. The first documented COVID-19 infection demonstrated at least 14 days after completion of the vaccination schedule was always evaluated in patients. SARS-CoV-2 virus infection was demonstrated in all patients using the RT-PCR (Real Time PCR) test. A positive result of the Rapid Antigen Test was always subsequently confirmed by the RT-PCR examination. A total of 65 patients were demonstrated in the monitored period. The available data were insufficient in 6 patients who were excluded from the analysis. All of the patients were identified based on the follow-up care at our workplace due to the treatment or follow-up care for haematology malignancy.

### 2.2. Vaccination and COVID-19 Treatment

The vaccination of the patients in the group was performed with vaccines approved for use in the Czech Republic in the monitored period—BNT162b2 (Comirnaty) manufactured by Pfizer/BioNTech, mRNA-1273 (Spikevax) manufactured by Moderna, or AZD1222 (Covishield/Vaxzevria) developed by AstraZeneca and the University of Oxford [13]. Therapy for COVID-19 infection was based on the severity of the condition on an outpatient basis or during hospitalisation at a COVID ward of the Olomouc University Hospital or local hospitals. The patients with proven infection were treated according to current treatment guidelines for COVID-19 therapy. Monoclonal antibodies, remdesivir, corticosteroids, molnupiravir, and supportive care, were used in therapy in accordance with current guidelines and the epidemiological situation [14,15]. The severity of the SARS-CoV-2 infection were classified according to WHO guidelines [14]. The actual haematological treatment and evaluation of the underlying disease was conducted according to the general guidelines of the Czech Haematological Society in accordance with international guidelines [16].

### 2.3. Clinical and Laboratory Data Assessment

Demographic data, the state of the underlying disease at the time of COVID-19 detection, anti-cancer therapy administered in the last three months, and the severity of the infection and its treatment were evaluated in the patient group. Furthermore, the laboratory findings were analysed, including selected blood count parameters (absolute white blood cell, lymphocyte and neutrophil count), C-reactive protein (CRP) level in the diagnosis of COVID-19 and the presence of IgG antibodies, at the earliest 1 month, after completion of vaccination and then after infection. The antibody response was monitored using the LIAISON^®^ SARS-CoV-2 S1/S2 IgG assay (DiaSorin) based on the chemiluminescence analysis (CLIA). COVID-19-related mortality in the group of patients was evaluated at the same time.

The data of all of the patients obtained from available medical documentation were processed anonymously, in accordance with the Code of Ethics of the Olomouc University Hospital and the Declaration of Helsinki. A total of 19 patients from the group were included in the EPICOVIDEHA study.

The data obtained were analysed and compared with our own historical cohort of 96 patients with haematologic malignancies and bone marrow failure syndromes (2 patients) who were treated for COVID-19 in the period from 1 March 2020 to 31 December 2020 and did not undergo vaccination. Detailed information about the cohort was published by Čerňan et al. [17].

### 2.4. Statistical Analysis

Statistical software IBM SPSS Statistics version 23 (IBM Corp., Armonk, NY, USA) was used for the data analysis. The quantitative parameters were compared using the Mann–Whitney U test, and the chi-square test was used to compare qualitative parameters. The pre-infection and post-infection antibody levels were compared using the Wilcoxon paired test. Data normality was evaluated using the Shapiro–Wilk test. All of the tests were performed at the significance level of 0.05.

## 3. Results

### 3.1. Study Cohort

The group consisted of a total of 59 patients with a median age of 64.5 (21.9–87.9) years. The group included 40 men and 19 women (ratio 2.1:1) with a median age of 64.4 (33.6–87.9) and 66.1 (21.9–87.4) years, respectively.

### 3.2. Underlying Haematological Disease

A total of 14 (23.7%) patients were treated for myeloproliferative malignancy, of whom four (6.8%) patients were treated for acute myeloid leukaemia (AML) or myelodysplastic syndrome (MDS). Three (5.1%) patients were treated for chronic myeloid leukaemia, two (3.4%) patients were treated for essential thrombocythaemia, and there was one (1.7%) case of chronic eosinophilic leukaemia in the group. Lymphoproliferative malignancies were the primary diagnosis in a total of 45 (76.3%) patients in the group. Fifteen (25.4%) patients were treated for chronic lymphocytic leukaemia (CLL) and non-Hodgkin’s lymphoma (NHL). Multiple myeloma was the primary diagnosis in six (10.2%) patients, and there was one case (1.7%) of the coincidence of multiple myeloma and NHL. Four (6.8%) patients were treated for Hodgkin’s lymphoma and a total of two (3.4%) patients for acute lymphoblastic leukaemia. There was also a case of AL amyloidosis and hairy cell leukaemia in the group (1.7%).

At the time of the demonstration of infection, a total of 27 (45.8%) patients in the monitored group were in complete remission of the underlying disease; 19 (70.4%) patients had been treated for lymphoproliferative diseases, and eight (29.6%) patients for myeloproliferative diseases. Partial remission was achieved in 14 (23.7%) patients and the best therapeutic response at the level of stable disease was demonstrated in two (3.4%) patients. Relapsed/refractory disease was present in six (10.2%) patients, and the achieved treatment response was not known in one patient (1.7%). In a total of nine (15.3%) patients, the underlying haematological disease was newly diagnosed, with no evaluation of the treatment response available at that time.

### 3.3. Anticancer Therapy

At the time of COVID-19 infection, a total of 48 (81.4%) patients received therapy for the underlying disease: 38 (79.2%) were treated for lymphoproliferation and 10 (20.8%) patients for myeloproliferation. A total of 11 (18.6%) patients had no anticancer therapy. A total of 21 (35.6%) patients were treated with chemotherapy and/or immunotherapy. A total of 16 patients received anti-CD20 monoclonal antibodies: rituximab was used in 15 cases and obinutuzumab in one case. Modern targeted oral therapy (so-called “small molecule drugs”) such as tyrosine kinase inhibitors (TKIs), *BCL2* and Bruton ‘s tyrosine kinase inhibitors was used in 12 (20.3%) patients in monotherapy and in combination with immunotherapy or chemotherapy in eight (13.6%) patients. A total of seven (11.8%) patients received other therapeutic modalities (growth factors, cyclosporin A or corticosteroid monotherapy).

### 3.4. COVID-19 Vaccination and Antibody Levels

A total of 48 (81.4%) patients underwent vaccination with the so-called mRNA vaccine—BNT162b2 (Comirnaty) or mRNA-1273 (Spikevax), and two (3.4%) patients were vaccinated with the so-called vector vaccine AZD1222 (Covishield/Vaxzevria). The type of vaccine was unknown in nine (15.3%) patients. A total of three (5.1%) patients in the group had a history of COVID-19 infection before vaccination, and 10 (16.9%) patients received the third dose (so-called booster) vaccine before documented COVID-19 infection in this study. COVID-19 infection after the second dose of the vaccine was documented after a median of 219 (14–341) days of vaccination in 48 patients with the available data. The median level of IgG antibodies in 22 vaccinated patients before infection was 4.34 (3.8–400) AU/mL, and the median level of antibodies with a history of infection was 110.5 (51.4–400) AU/mL in 14 patients with the available data. Pairwise comparison of antibody levels before and after infection showed that patients had significantly higher antibody levels after COVID-19 infection (*p* = 0.012).

### 3.5. Course and Therapy of COVID-19

SARS-CoV-2 virus typing demonstrated the delta variant in 10 (90.9%) out of 11 patients with the available data. The omicron variant was demonstrated only in one case, in January 2022, before the end of the monitored period. Selected laboratory parameter levels used for the diagnosis of COVID-19 are shown in Table 1. The course of SARS-CoV-2 infection was evaluated as asymptomatic in eight (13.6%) patients, mild in 30 (50.8%) and moderate in 15 (25.4%). In six (10.2%) patients, the course was evaluated as severe to critical. COVID-19 specific therapy was used in a total of 30 (50.8%) patients. Four (6.8%) patients received remdesivir, while one (1.7%) patient also used so-called monoclonal antibodies at the same time. Of the total number of 24 patients who received monoclonal antibodies, casirivimab-imdevimab was administered in 14 (58.3%) and bamlanivimab-etesevimab in six (25.0%) patients. No data on the administered antibody type was available in four (16.7%) patients. Three (5.1%) patients received molnupiravir. A total of 29 (49.2%) patients had no specific antiviral therapy for COVID-19. Convalescent plasma was not used in any patient. A total of nine (15.3%) patients in the group required oxygen support during COVID -19 therapy. Oxygen mask or cannula support was sufficient in five (8.5%) patients; four (6.8%) required high-flow oxygen therapy (HFOT) or artificial pulmonary ventilation. Therapy during hospitalisation was required for a total of 16 (27.1%) patients in the group. The duration of hospitalisation in 15 patients, with the available data, ranged from 2 to 25 (median 10) days. A total of three (5.1%) patients in the group died as a result of SARS-CoV-2 virus infection. Out of this group, two patients were treated for CLL and one for multiple myeloma.

### 3.6. Comparison with a Historical, Non-Vaccinated Cohort of Patients

The group of non-vaccinated patients consisted of a total of 96 patients; 2 patients in the group were treated for bone marrow failure syndrome. The compared groups did not differ significantly in age (median 64.5 vs. 66.0 years, *p* = 0.771) or representation of lymphoproliferative (76.3% vs. 67.7%, *p* = 0.254) or myeloproliferative malignancies (23.7% vs. 30.2%, *p* = 0.382). In the group of vaccinated COVID-19 patients, the proportion of men was significantly higher (67.8% vs. 41.7%, *p* = 0.002). Although a significantly larger proportion of patients had a primary haematological disease in complete remission in the group of vaccinated patients (45.8% vs. 28.1%, *p* = 0.025), representation of patients undergoing therapy for the primary disease was comparable (81.4% vs. 78.1%, *p* = 0.629). Blood count parameters (total white blood cell count, absolute lymphocyte and neutrophil counts) and CRP level at the diagnosis of COVID-19 showed no significant differences in the two monitored cohorts (*p* > 0.05).

Patients in the vaccinated group were significantly more likely to have an asymptomatic to moderate course of COVID-19 according to the WHO classification (89.9% vs. 68.6%, *p* = 0.003); severe and critical course was significantly less common in this group (10.2% vs. 31.4%, *p* = 0.003). A severe to critical course of COVID-19 was less common in patients with underlying disease in complete remission compared to patients not achieving complete remission in non-vaccinated cohort (4/27, 14.8%, and 23/69, 33.3%, respectively). Chronic lymphocytic leukeamia was the underlying disease in 2 (50.0%), respectively in 7 (30.4%) patients in mentioned groups. Only one vaccinated patient with CLL in complete remission had critical course of COVID-19, but 5/31 (16.1%) patients not achieving complete remission in the vaccinated group had severe to critical course of infection. Interestingly, out of this group, four of the patients had CLL.

The need for hospitalisation was significantly higher in non-vaccinated patients (72.6% vs. 27.1%, *p* < 0.0001) and the duration of hospitalisation was significantly longer in these patients (median 14 vs. 10 days, *p* = 0.045). The need for oxygen therapy with an oxygen mask/cannula was insignificantly lower in vaccinated patients (8.5% vs. 18.9%, *p* = 0.076). Artificial pulmonary ventilation or HFOT was used in an insignificantly smaller number of patients in the vaccinated group (6.8% vs. 13.7%, *p* = 0.184). The need for remdesivir, as indicated in patients with COVID-19 and the need for oxygen therapy, was also insignificantly lower in this group (6.8% vs. 13.7%, *p* = 0.054).

**Figure 1:**
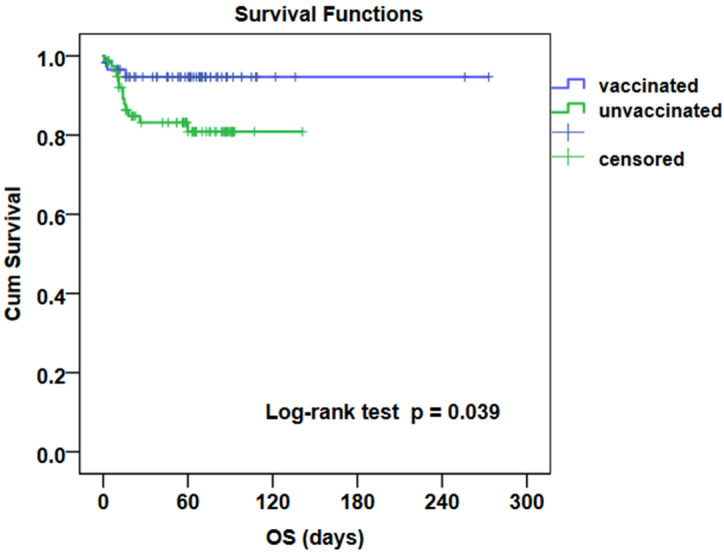
Overall survival in compared groups of patients with vaccination or without vaccination. OS—overall survival.

Kaplan-Meier analysis with the Log-rank test demonstrated significantly better survival in the group of vaccinated patients (*p* = 0.039). COVID-19 mortality was significantly higher in the group of patients who did not undergo vaccination (15.6% vs. 5.1%, *p* = 0.047). Table 1 clearly shows the differences in compared parameters in the presented group of patients and the historical group of patients without vaccination. Figure 1 shows overall survival in compared groups of patients with vaccination and without vaccination.

## 4. Discussion

The paper brings an analysis of a group of 59 patients with haematologic malignancies, with breakthrough SARS-CoV-2 infection documented despite vaccination. Part of the paper also compares the course of COVID-19 with our own historical cohort of non-vaccinated patients. The presented results offer a unique comparison of two cohorts treated at one specialised haematological care centre and are also the first publication focused on breakthrough infections in vaccinated patients with haematologic malignancies from the Czech Republic. Evaluated populations have a similar age structure (64.5 vs. 66.0 years, *p* = 0.771) and the proportion of lymphoproliferative and myeloproliferative diseases, respectively (76.3% vs. 67.7%, *p* = 0.254 and 23.7% vs. 30.2%, *p* = 0.382, respectively). There were also two patients with bone marrow failure syndromes in the group of non-vaccinated patients, making up only 2/96 (2.1%) of the total number of patients. Vijenthira et al. reported in their published study that the risk of COVID-19-related death in this group of patients was comparable to haematological malignancies; therefore, these patients were not excluded from the evaluation [6]. Consistent with previously published results, malignant lymphoproliferative disorders represent a predominant group of diagnoses in both cohorts [5]. A significantly larger proportion of patients in the vaccinated group achieved complete remission of the primary disease (45.8% vs. 28.1%, *p* = 0.025). An explanation may lie in the trend of patient vaccination at the time of completion of anticancer therapy due to the achievement of the complete remission of the disease.

The history of COVID-19 infection led to an increase in antibody levels in the subgroup of vaccinated patients (*p* = 0.012). Since only a small part of patients had a history of infection before vaccination (3/59, 5.1%) and only 10 (16.9%) patients received the third dose of the vaccination, we can conclude that infection after vaccination has a similar effect in the group as administration of the so-called booster dose. In contrast, Thakkar et al. demonstrated that also patients with a history of COVID-19 produced higher levels of antibodies after subsequent vaccination (46.7 vs. 5.3 AU/mL, *p* < 0.001) [8]. Both our compared cohorts had a significant representation of patients with CLL and B-NHL (31/59, 52.5%, and 44/96, 46.8%, respectively), which may have affected antibody levels due to already known worse seroconversion after vaccination [8,18,19].

In addition to the antibody response, which may be insufficient, especially in patients treated for B lymphoproliferation with anti-CD20 antibodies, T-cell-mediated cellular immunity is an important part of the body’s immune response to COVID-19. Stimulation of the T cell response after vaccination is often an underestimated effect of vaccination in patients who have not achieved seroconversion after vaccination [19]. Pagano et al. did not observe a significant difference in mortality in subgroups of vaccinated patients who responded or did not respond to seroconversion or received one or two doses according to the vaccination schedule. Thus, the value of post-vaccination antibodies cannot be considered a single factor [11]. However, a test evaluating the cellular component of post-vaccination immunity is not yet available in the routine diagnosis. Furthermore, the use of a different booster vaccination than the product used within the initial vaccination scheme, even though not utilised in any of the patients in our cohort vaccinated with the third dose, still remains a controversial question [20,21].

Previously published studies have suggested that patients with CLL and NHL, often treated with anti-CD 20 antibodies, are the most at-risk population [18,19]. A more common severe to critical course of COVID-19 was seen in patients not achieving complete remission of haematologic malignancy in both vaccinated and non-vaccinated cohorts in our study. Chronic lymphocytic leukaemia was the most common diagnosis in our patients with severe infection. A study by Pagano et al. in patients who developed COVID-19 after vaccination also showed higher mortality in this group of patients, in contrast to previous results, where the highest mortality rate in non-vaccinated patients was reported in AML and MDS patients [5,11]. In contrast, studies have demonstrated a good antibody response to vaccination in AML patients, which may explain the differences in the above-stated papers [10,19].

Our study demonstrated a significant positive effect of vaccination on the course of the disease and the need and duration of hospitalisation (*p* < 0.05). The need for oxygen therapy with an oxygen mask/cannula or HFOT/APV was insignificantly lower in vaccinated patients. Although it is now clear that vaccination cannot prevent the SARS-CoV-2 disease in 100% of cases, it led in the presented paper to a significant reduction in infectious mortality in the group of patients with haematologic malignancies (5.1% vs. 15.6%, *p* = 0.047). The effect of vaccination on reducing COVID-19 mortality in patients with haematological malignancies has been demonstrated even in other studies [11,12]. However, compared to the healthy population, there is an increased risk of breakthrough infections in patients with haematological malignancies, with a severe course and high mortality despite vaccination [9,12]. Therefore, other anti-epidemiological measures, such as wearing respirators and social isolation in high-risk patients, should not be neglected.

An important factor for the evaluation of the differences between the two groups is also the different time periods of follow-up and the resulting different variants of the virus with different biological behaviour. The SARS-CoV-2 omicron variant shows a higher infectious rate but a lower predisposition to a clinically severe course [22]. The delta variant dominated Europe in the second half of 2021 [23]. The delta variant was confirmed in up to 10 of the 11 patients in our study who underwent vaccination and virus typing. The omicron variant was demonstrated in one case in this study, which may be related to the monitored period ending on 31 January 2022. Pagano et al. observed lower mortality in their study in the 2nd pandemic wave (October–December 2020) as compared to the 1st wave (March–May 2020), 40.7 vs. 24.8%, *p* < 0.0001 [5]. This may be explained by our better understanding of the biology of the viruses and improved diagnostic and therapeutic procedures with the implementation of new drugs in clinical practice. Another important aspect in the evaluation of the differences in the monitored groups was the availability of monoclonal antibodies, effective, especially against the delta variant of the virus. A total of up to 40.1% of our patients received monoclonal antibodies. Weinbergerová et al. showed in their study that the administration of monoclonal antibodies resulted in a significant reduction in the probability of a complicated course of the disease [7].

The limitation of the study is its retrospective character, a limited number of enrolled patients, missing data, and new therapeutic modalities and variants of the virus with different biological behaviour appearing over time. Further research is needed to better understand the virus and its new variants and to develop effective booster vaccines or antivirals or monoclonal antibodies for preexposure prophylaxis with regard to a high risk of a complicated COVID-19 course in patients with haematologic malignancies despite previous vaccination. A reduction in mortality in the most at-risk population of patients with haematologic malignancies may be expected only in this way.

## 5. Conclusions

Patients with haematologic malignancies who were vaccinated against COVID-19 had a less severe clinical course of breakthrough infection, with a lower need for hospitalisation and lower mortality, compared to a historical cohort of non-vaccinated patients. Vaccination against COVID-19 further remains the basic preventive tool for the most vulnerable groups of patients. Further development of effective vaccines and new treatment modalities are essential for the management of the COVID-19 pandemic.

## Figures and Tables

**Table 1:** Comparison of selected parameters in groups of patients with vaccination and without vaccination.

Parameter	Non-Vaccinated (96 Patients)		Vaccinated (59 Patients)		*p*
Age—median (min-max) years	66.0 (26–84)		64.5 (22–88)		0.771
Men—number	40/96 (41.7%)		40/59 (67.8%)		0.002
Lymphoproliferation	65/96 (67.7%)		45/59 (76.3%)		0.254
Myeloproliferation	29/96 (30.2%)		14/59 (23.7%)		0.382
Primary disease in CR	27/96 (28.1%)		27/59 (45.8%)		0.025
Active haematological therapy	75/96 (78.1%)		48/59 (81.4%)		0.629
Asymptomatic to moderate course of COVID-19	59/86 (68.6%)		53/59 (89.8%)		0.003
Severe and critical course of COVID-19	27/86 (31.4%)		6/59 (10.2%)		0.003
Need for oxygen therapy—mask/cannula	95 items	18/95 (18.9%)	59 items	5/59 (8.5%)	0.076
Need for APV/HFOT	95 items	13/95 (13.7%)	59 items	4/59 (6.8%)	0.184
Administration of remdesivir	17/96 (17.7%)		4/59 (6.8%)		0.054
Need for hospitalisation	69/95 (72.6%)		16/59 (27.1%)		<0.0001
Duration of hospitalisation Median (min-max) days	47 items	14 (2–67)	15 items	10 (2–25)	0.045
Baseline CRP level Median (min-max) mg/L	60 items	31.9 (0.2–299)	31 items	34.04 (4–204)	0.903
Baseline WBC level Median (min-max) 10^9^/L	61 items	5.23 (0.03–145.4)	31 items	5.98 (1.89–54.2)	0.306
Baseline LY level Median (min-max) 10^9^/L	59 items	0.91 (0.00–139.7)	31 items	1.31 (0.17–46.5)	0.225
Baseline ANC level Median (min-max) 10^9^/L	59 items	3.40 (0.00–48.41)	31 items	2.97 (0.99–7.13)	0.845
COVID-19 mortality	15/96 (15.6%)		3/59 (5.1%)		0.047

CR—complete remission, APV—artificial pulmonary ventilation, HFOT—high flow oxygen therapy, CRP—C-reactive protein, WBC—white blood cells, LY—lymphocytes, ANC—absolute neutrophil count.

## Data Availability

All data presented in this study are included in this article.
